# Aortitis as a High-Risk Vascular Syndrome: Integrating Phenotype-Driven Diagnosis, Multidisciplinary Assessment, and Personalised Management

**DOI:** 10.3390/jpm16080430

**Published:** 2026-08-14

**Authors:** Georgios P. Georghiou, Klitia Socratous, Sotiris Kyriakou, Konstantinos Lampropoulos, Panos Georghiou, Amalia Georgiou, Marilina Neokleous, Iakovos Ttofi, Nikolas Iosif, Filippos Triposkiadis

**Affiliations:** 1 Department of Surgery, Gray Faculty of Medical & Health Sciences, Tel Aviv University, Tel Aviv 6997801, Israel; 2 School of Medicine, European University Cyprus, Egkomi, Nicosia 2404, Cyprus; ks202091@students.euc.ac.cy (K.S.); k.lampropoulos@euc.ac.cy (K.L.); 2024euc3099@students.euc.ac.cy (I.T.); f.triposkiadis@external.euc.ac.cy (F.T.); 3 Barts and The London School of Medicine and Dentistry, Queen Mary University of London, London E1 2AD, UK; s.kyriakou@nhs.net (S.K.); p.georghiou@smd21.qmul.ac.uk (P.G.); 4 Department of Cardiology, Krankenhaus Maria-Hilf, 47805 Krefeld, Germany; a.georgiou@alexianer.de; 5 School of Medicine, University of Cyprus, Aglanztia, Nicosia 2029, Cyprus; mneokl01@ucy.ac.cy; 6 Klinik für Gastroenterologie, Hepatologie, Endokrinologie, Diabetologie, Helios Universitätsklinikum, 42283 Wuppertal, Germany; nikolas.iosif@helios-gesundheit.de

**Keywords:** aortitis, large-vessel vasculitis, giant cell arteritis, Takayasu arteritis, infectious aortitis, IgG4-related disease, multimodality imaging, multidisciplinary care, personalised medicine, aortic aneurysm

## Abstract

Aortitis—inflammation of the aortic wall—presents at the interface of vasculitis, infection, structural aortic disease, and cardiovascular risk. It may occur in giant cell arteritis (GCA), Takayasu arteritis, immunoglobulin G4 (IgG4)-related disease, drug-induced injury, infection, or as an isolated finding after aortic surgery. Modern imaging detects aortic inflammation more frequently, but the main challenge is classification rather than detection: determining whether disease is infectious or immune-mediated, active or dominated by fixed structural damage, systemic or isolated, and whether the dominant threat is aneurysm, dissection, undertreated infection, or avoidable immunosuppression. This review considers aortitis as a high-risk vascular syndrome requiring aetiology-first classification rather than descriptive labelling. Before escalating immunosuppression, infection must be actively excluded and inflammatory activity distinguished from fixed vascular damage. Treatment should be individualised according to phenotype, age, vascular territory, comorbidity, and toxicity risk, with surveillance continuing even after symptoms and inflammatory markers improve. Optimal care depends on multidisciplinary assessment integrating rheumatology, infectious diseases, vascular surgery, radiology, and cardiology expertise. Progress will require standardised imaging definitions, registries linking inflammatory control with structural vascular outcomes, and validation of artificial intelligence (AI) tools before clinical adoption.

## 1. Introduction

Aortitis is often introduced as inflammation of the aortic wall—a simple anatomical term. In practice, however, the label is rarely simple, as the same radiological or histological finding may reflect giant cell arteritis (GCA), Takayasu arteritis, IgG4-related disease, immune-checkpoint inhibitor (ICI) toxicity, granulocyte-colony stimulating factor (G-CSF) exposure, or a localised inflammatory lesion identified only after thoracic aortic surgery [1,2,3,4,5]. Rather than a final diagnosis, aortitis is better understood as a warning signal requiring clarification of cause, inflammatory activity, anatomical distribution, structural risk, and treatment implications.

The stakes are high because the aorta has little tolerance for diagnostic delay. Ongoing inflammation may cause mural thickening, fibrosis, elastic fibre fragmentation, and matrix degradation. These changes may progress to aneurysm formation, dissection, or rupture, while branch-vessel stenosis and aortic regurgitation develop alongside systemic complications including coronary or cerebral ischaemia, renovascular hypertension, and limb claudication that necessitate repeated vascular procedures [1,2,6,7,8]. However, early presentation is often non-specific, such as fever, malaise, weight loss, fatigue, chest or abdominal discomfort, and raised inflammatory markers may be attributed to infection, malignancy, polymyalgia rheumatica, degenerative aortic disease, or postoperative inflammation. By the time pulse deficits, bruits, blood pressure asymmetry, organ ischaemia, aneurysm, or dissection become evident, irreversible vascular damage may already have occurred.

A major difficulty is that aortitis rarely fits within a single specialty. Rheumatologists usually approach it through large-vessel vasculitis, whereas infectious diseases physicians focus on microbial seeding, source control, and antimicrobial treatment. Radiologists characterise wall thickening, metabolic uptake, and structural complications, and surgeons may first encounter the disease as unexpected inflammation during aortic repair. Cardiologists manage blood pressure control, valve complications, and long-term cardiovascular risk. Each perspective brings essential expertise, although none alone provides a complete picture of the disease.

This review frames aortitis as a high-risk vascular syndrome requiring cause-first classification, distinction between active inflammation and established damage, individualised treatment, and long-term surveillance. Management should be guided not only by diagnostic label but by vascular anatomy, infection risk, inflammatory activity, structural vulnerability, treatment toxicity, comorbidity, and patient priorities. A 75-year-old patient, for example, with GCA-related aortitis, diabetes, osteoporosis, and expanding aneurysm requires different decisions than a 30-year-old patient with Takayasu arteritis, critical stenosis, and preserved renal function. Both have “aortitis”, but they do not have the same syndrome.

## 2. Review Scope and Approach

A structured narrative search of PubMed/MEDLINE was conducted from database inception to 23 July 2026. The core Boolean syntax was: (“aortitis” OR “large-vessel vasculitis” OR “giant cell arteritis” OR “Takayasu arteritis” OR “IgG4-related aortitis” OR “infectious aortitis” OR “clinically isolated aortitis”) AND (“imaging” OR “treatment” OR “biologic therapy” OR “surgery” OR “surveillance” OR “outcomes” OR “artificial intelligence” OR “radiomics”). English-language guidelines, randomised trials, prospective and retrospective cohorts, systematic reviews and clinically informative case series were considered; isolated case reports were retained only for rare infectious or drug-associated phenotypes. Studies without aortic or large-vessel relevance, conference abstracts without full methods and duplicate reports were excluded. Reference lists of major guidelines and reviews were screened for additional studies. Because this was a narrative rather than systematic review, no PRISMA screening flow, formal study-count denominator, risk-of-bias assessment or meta-analysis was undertaken.

The evidence base is uneven, with practical consequences for care. Randomised evidence supports several remission and glucocorticoid-sparing strategies in GCA, but evidence is limited for clinically isolated aortitis, Takayasu arteritis, infectious aortitis and surgically discovered disease. Several recommendations therefore rely on observational data and expert consensus, or on extrapolation from large-vessel vasculitis guidance [9,10,11] and general aortic disease guidance [7]. Accordingly, “proposed”, “pragmatic” and “reasonable” in this review denote author interpretation rather than a validated universal recommendation.

## 3. Aortitis Is a Syndrome, Not a Single Disease

While distinguishing infectious from non-infectious disease is essential, this distinction alone does not guide treatment. Infectious aortitis, though uncommon, is immediately dangerous and may arise through haematogenous spread, septic embolisation, contiguous infection, infection of atherosclerotic plaques or aneurysms, or seeding of a previously injured aortic segment [7,12,13]. The pathogen spectrum is also broad, encompassing Salmonella species, Staphylococcus aureus, Streptococcus species, and other Gram-negative organisms, alongside infections caused by syphilis, tuberculosis, Q fever, and fungal agents [7,12,13]. Regardless of the organism, management depends on prompt recognition, microbiological sampling, targeted antimicrobial therapy, and early vascular surgical evaluation in the presence of aneurysm, pseudoaneurysm, dissection, rupture, or uncontrolled sepsis [7,12,13]. Contemporary consensus documents classify infective aortic disease principally by clinical, microbiological and anatomical features [14]. HIV and hepatitis testing may be clinically indicated during evaluation, but these serologies should not be presented as common parallel causes of primary viral aortitis.

Reports of less common pathogens reinforce the same principle, showing that infectious aortitis requires prompt recognition and source control while its presentation may vary considerably. Escherichia coli infection may manifest with periaortic fluid, pseudoaneurysm formation, perforation, or delayed abscess development. Burkholderia pseudomallei endograft infections underscore how geography, environmental exposure, antimicrobial resistance patterns, and source control intersect in complex ways. Listeria monocytogenes periaortitis can remain culture-negative, only becoming apparent through tissue sampling or molecular diagnostics, while hypervirulent Klebsiella pneumoniae strains can cause metastatic infection with mycotic aneurysm formation [15,16,17,18]. Clinically, these examples show that infectious aortitis behaves differently and should not be treated simply as a vasculitis mimic. It progresses differently, requires different decisions, and carries different consequences if missed.

When infection has been excluded, non-infectious aortitis is usually considered within the framework of large-vessel vasculitis. GCA usually affects patients over 50 years of age and may involve cranial vessels, the aorta and its major branches. Takayasu arteritis typically presents earlier in life, has a strong female predominance and frequently involves the aortic arch and its branches, with stenosis, occlusion, aneurysm or renovascular hypertension [19,20,21,22,23,24,25]. These patterns are helpful, but they should not be applied rigidly. GCA can present predominantly with extracranial large-vessel disease and few cranial symptoms and Takayasu arteritis may be diagnosed only after vascular damage has developed. Histological aortitis may also be found in resected aortic tissue without an obvious systemic syndrome [3,26,27]. Takayasu arteritis has recognised genetic associations, notably HLA-B*52 in several populations, although this marker is neither diagnostic nor suitable for routine screening [28]. Because onset commonly occurs during the reproductive years, pre-conception counselling and coordinated rheumatology, maternal-medicine and cardiovascular follow-up are important; pregnancy is associated particularly with hypertensive complications [29]. Nationwide data also confirm that patients with GCA remain at increased risk of thoracic aortic complications [30].

Beyond the major large-vessel vasculitides, other immune-mediated and inflammatory aetiologies complicate the differential diagnosis. IgG4-related aortitis and periaortitis may cause adventitial or periaortic fibroinflammatory thickening, aneurysm, retroperitoneal fibrosis or multi-organ disease [31,32]. Behcet disease, systemic lupus erythematosus, sarcoidosis, spondyloarthritis and other systemic inflammatory disorders may involve the aorta. Drug-associated aortitis with G-CSF and ICI is recognised and challenges the older binary distinction between idiopathic autoimmunity and infection [33,34,35].

Among these entities, IgG4-related aortitis requires careful diagnostic assessment. Serum IgG4 alone is neither sensitive nor specific enough to define the diagnosis, and IgG4-positive plasma cells in tissue must be interpreted in clinicopathological context. A defensible diagnosis requires coherent imaging, evidence of organ involvement where present, supportive histology such as IgG4-rich lymphoplasmacytic inflammation, storiform fibrosis or obliterative phlebitis when available, exclusion of infection and malignancy, and longitudinal response to treatment [31,32]. This matters because relapse risk, surveillance and steroid-sparing strategies may differ from those used in GCA or Takayasu arteritis [36]. In a prospective single-centre IgG4-related disease cohort, 89 of 587 patients (15.2%) had aortitis/periaortitis or periarteritis; most lesions involved the abdominal aorta or iliac arteries, and radiological improvement was common after glucocorticoid-based treatment. These observational data do not establish an optimal regimen [37].

The entity of clinically isolated aortitis illustrates the difficulty of rigid disease classification. Some patients have histological aortitis unexpectedly identified during surgery for thoracic aneurysm or dissection [3,26,27]. Some remain without systemic disease, whereas others later develop GCA, polymyalgia rheumatica, another rheumatic diagnosis, new vascular lesions or further need for intervention [3,26,27,38,39,40]. These findings are not incidental; they suggest that localised aortic inflammation at presentation may still have prognostic consequences. At the time of surgery, the patient may not fit a conventional vasculitis category and may still need full vascular mapping and long-term follow-up. Surgical series identify inflammatory aortitis in approximately 2–12% of thoracic aortic operations; one 1119-case cohort found 41 cases (3.7%). In a separate cohort, 45% of clinically isolated aortitis patients developed new radiographic lesions and 40% underwent further vascular surgery during a mean 56.2-month follow-up [38,39]. A matched cohort of 53 patients with clinically isolated aortitis and 109 comparators found more thoracic aortic branch-vessel abnormalities but no excess new or worsening aortic aneurysms or surgical complications, despite most patients not receiving immunosuppression [40]. The principal demographic, anatomical, clinical and therapeutic distinctions between giant cell arteritis and Takayasu arteritis are summarised in Table 1.

The practical question is therefore not only “Is there aortitis?”; clinicians also need to establish the most likely cause, whether infection has been reasonably excluded, whether the process is active or not, which aortic segments and branch vessels are involved, whether aneurysm, stenosis, dissection, valve disease or rupture risk is present, whether systemic disease is evident, what treatment risk is acceptable, and what surveillance is required after symptoms improve. This sequence makes the label clinically useful.

**Figure 1:**
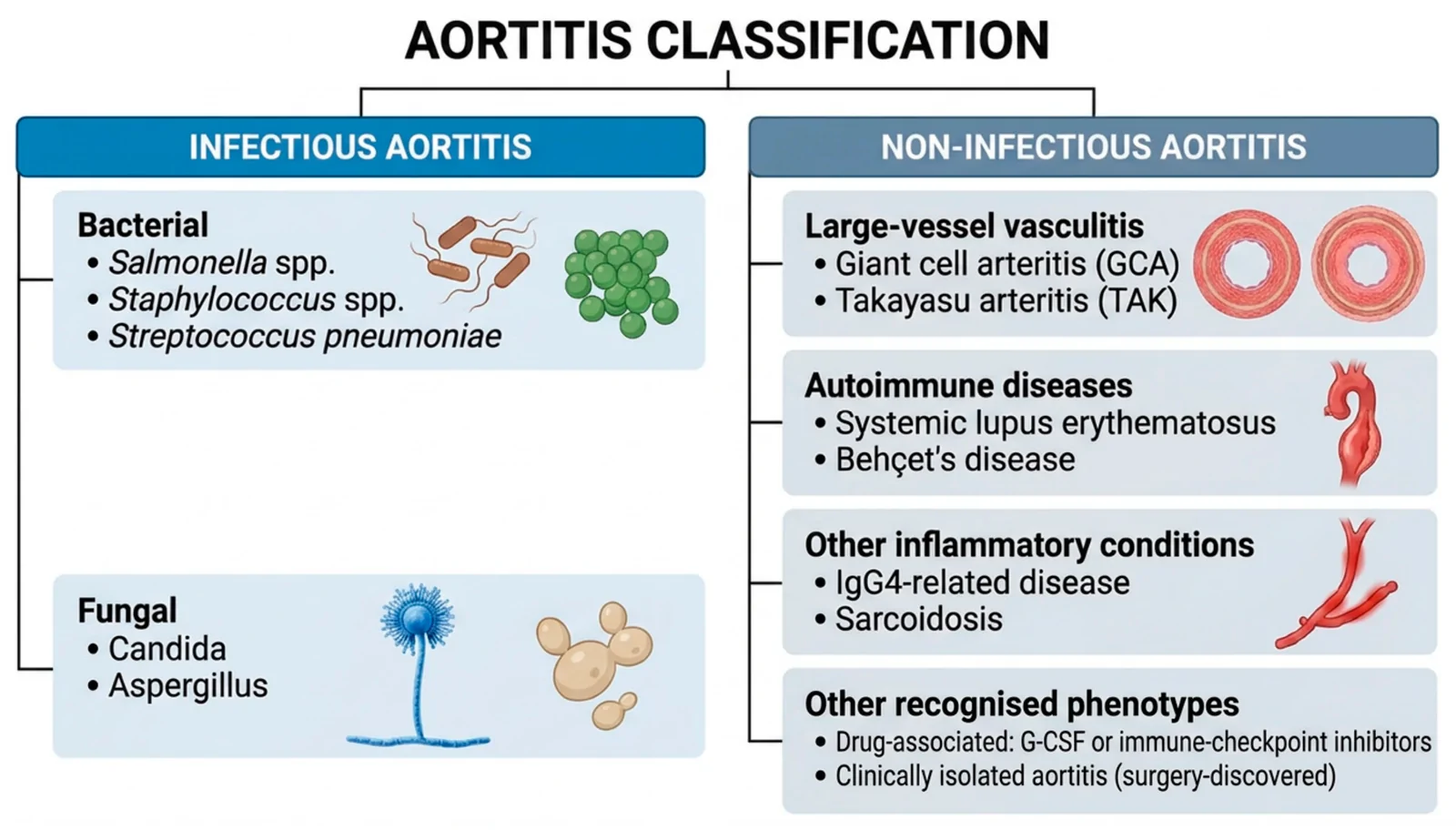
Classification of aortitis according to infectious, non-infectious, drug-associated and clinically isolated phenotypes. The figure highlights that aortitis is a syndrome requiring aetiology-first classification rather than descriptive labelling alone. Abbreviations: GCA, giant cell arteritis; G-CSF, granulocyte-colony stimulating factor; ICI, immune-checkpoint inhibitor; IgG4, immunoglobulin G4; TAK, Takayasu arteritis.

The major clinical categories of aortitis are summarised in Figure 1.

## 4. Clinical Presentation: Why Diagnosis Is Often Delayed

Aortitis is often diagnosed late because it rarely presents with a single pathognomonic symptom. Early in the disease course, patients may experience non-specific constitutional symptoms including fever, night sweats, malaise, fatigue, anorexia, and weight loss, alongside pain affecting the chest, back, abdomen, or joints. Laboratory evaluation typically reveals raised inflammatory markers, but these findings remain non-specific and may equally suggest infection, malignancy, systemic autoimmune disease, degenerative vascular pathology, or postoperative inflammation [1,2]. In older patients, headache, scalp tenderness, jaw claudication, visual symptoms or polymyalgia rheumatica may point toward GCA, but large-vessel disease may occur with minimal cranial involvement [19,20,24,25]. In younger patients, Takayasu arteritis may begin with constitutional symptoms before limb claudication, reduced pulses, bruits, blood-pressure discrepancies, hypertension, cerebrovascular symptoms or renal artery disease become apparent [21,22,23].

Clinical examination remains underused, although simple bedside findings, including asymmetrical or absent pulses, inter-limb blood pressure discrepancies, vascular bruits over major arteries, signs of aortic regurgitation, limb ischaemia, or unexplained hypertension in the context of systemic inflammation, can substantially narrow the differential diagnosis before any imaging is performed. Aortitis should enter the differential when an aneurysm appears at an atypical age, expands unexpectedly rapidly, displays periaortic inflammatory changes, develops after recurrent vascular procedures, accompanies otherwise unexplained systemic inflammation, or demonstrates wall thickening out of proportion to atherosclerotic burden.

The absence of these signs does not exclude aortitis, as aortic inflammation may remain silent until aneurysm, dissection, rupture, stenosis or embolisation occurs. Wider access to computed tomography angiography (CTA), magnetic resonance angiography (MRA), vascular ultrasound scan (USS) and 18F-fluorodeoxyglucose positron emission tomography (FDG-PET) has improved detection of subclinical large-vessel disease, but it has also created a new problem as clinicians now see more abnormal aortas than they can confidently classify [10,44,45,46,47].

The consequences of delayed recognition are tangible and serious. A patient with systemic symptoms may receive empirical treatment and later present with an aneurysm. Another patient with infectious aortitis may receive corticosteroids for presumed vasculitis before blood cultures, tissue sampling or source control have been pursued. A third may undergo aortic surgery, receive a pathology report showing aortitis and then leave hospital without a structured follow-up plan. These failures are clinically likely consequences of fragmented pathways.

## 5. Pathobiology: Active Inflammation, Infection and Established Damage

Distinguishing active inflammation from established structural damage is clinically critical. Active inflammation reflects ongoing immune or microbial injury that may respond to immunosuppression, antimicrobial therapy, source control or treatment of an underlying disease. Established structural damage includes fibrosis, medial degeneration, elastic lamina disruption, calcification, aneurysm, stenosis, occlusion, dissection or postoperative change that can persist after inflammation has become clinically quiet. The distinction is imperfect in practice, but it directly affects whether clinicians escalate medication, operate or observe.

In large-vessel vasculitis, immune activation appears to originate in the adventitia and vasa vasorum, where dendritic cells activate and recruit T lymphocytes and macrophages, triggering a cascade of cytokine amplification, granulomatous inflammation, vascular smooth muscle dysfunction, neoangiogenesis, and progressive matrix degradation [48,49,50,51]. Key pathways implicated in this process include interleukin-6 (IL-6), IL-17, interferon-gamma, granulocyte-macrophage colony-stimulating factor, and matrix metalloproteinases [48,49,50,51]. Histological examination typically reveals infiltration by lymphocytes and macrophages, with multinucleated giant cells forming granulomas with scarring and disrupted elastic laminae [26,27,48]. Chronic disease may progress to fibrosis and loss of elasticity even when systemic inflammation seems controlled.

GCA and Takayasu arteritis share several immunopathological themes, but their clinical trajectories differ. Takayasu arteritis often causes wall thickening, stenosis, occlusion, aneurysm and vascular insufficiency in younger patients [21,22,23]. GCA may present with cranial ischaemia, polymyalgia or systemic symptoms, while aortic dilatation and aneurysm may develop later [8,19,20,24,25]. The main danger is assuming that clinical remission means the aorta is structurally safe as aortitis can leave a structurally vulnerable aortic wall long after fever, pain and C-reactive protein (CRP) levels have improved.

In contrast to immune-mediated disease, infectious aortitis follows a different pathobiological logic. Microbial invasion of a predisposed or damaged aortic wall may rapidly cause suppurative inflammation, necrosis, pseudoaneurysm, rupture and death [7,12,13,14]. Susceptibility is increased by pre-existing atherosclerotic plaque or aneurysm, prosthetic material, previous vascular intervention, systemic immunosuppression and ongoing bacteraemia. In this setting, the principal therapeutic error is premature immunosuppression. Fever, bacteraemia, endocarditis risk, recent infection, prosthetic material or high-risk anatomy should therefore prompt active infection exclusion before wall thickening or FDG uptake is attributed to vasculitis.

Treatment can uncouple biomarkers from vascular biology: glucocorticoids and IL-6 blockade may normalise CRP levels before disease control, whereas persistent CTA or MRA abnormalities may represent inactive damage. Activity should therefore be judged from concordant clinical, laboratory and serial imaging evidence; no single validated test separates active inflammation, infection and fixed damage in every patient [9,10,44,51].

**Figure 2:**
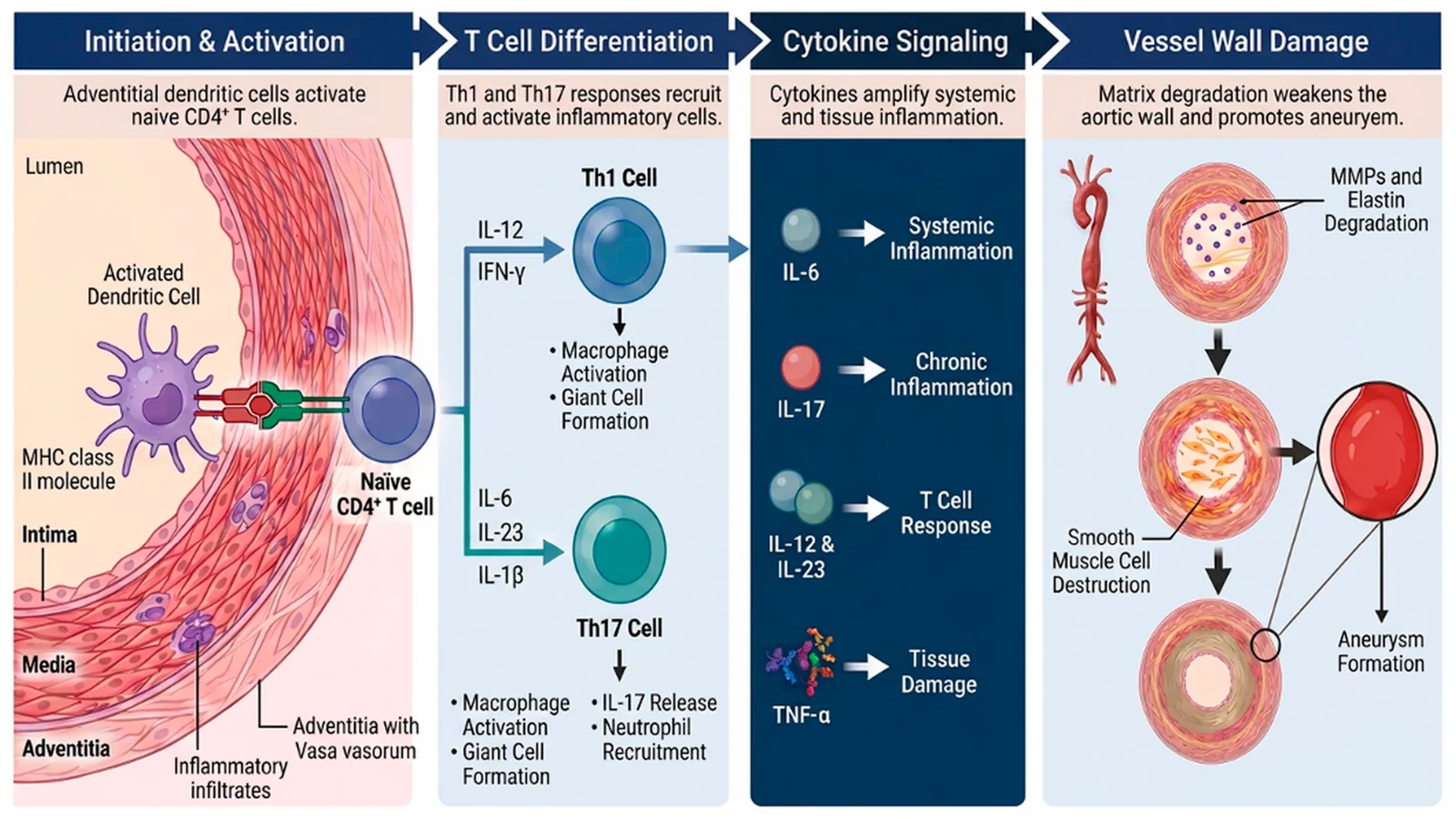
Pathophysiology of aortitis. The illustrated immune-mediated pathway links adventitial dendritic-cell activation and T-cell differentiation with cytokine amplification, inflammatory-cell recruitment, smooth-muscle injury, extracellular-matrix degradation and progressive structural complications, including aneurysm formation. Abbreviations: CD4+, cluster of differentiation 4-positive; IFN-γ, interferon gamma; IL, interleukin; MHC, major histocompatibility complex; MMP, matrix metalloproteinase; Th1/Th17, T-helper 1/17; TNF-α, tumour necrosis factor alpha.

The main pathobiological mechanisms linking inflammation, infection and structural vascular damage are shown in Figure 2.

## 6. Diagnosis: Assembling Evidence from Imperfect Tools

Diagnosis begins with suspicion, but no single history item, laboratory result, imaging sign or histological feature is sufficient across all patients. Initial clinical evaluation should explore a broad range of features including constitutional symptoms, cranial manifestations suggestive of GCA, polymyalgia, limb claudication, neurological deficits, and evidence of renal or mesenteric ischaemia. The history should document chest or abdominal pain, aortic valve dysfunction, recent infections, or endocarditis risk, alongside details of immunosuppression, vascular grafts, previous aortic surgery, or recent cancer therapy including ICI or G-CSF. Associated autoimmune features, oral or genital ulceration, and relevant family history should complete the assessment [1,2,3,4,5,33,34,35].

Diagnostic evaluation must account for patient-specific variables that alter both disease probability and test interpretation. For example, age influences differential diagnosis as GCA is rare below 50 years while Takayasu arteritis is uncommon beyond 40 years. Sex, ethnicity, and geographic origin may also affect disease prevalence. Immunosuppression, cancer therapy, vascular grafts, and recent infection increase the probability of infectious aortitis, and diabetes, chronic kidney disease, and osteoporosis increase steroid toxicity risk and influence treatment decisions even before final diagnosis. A diagnostic pathway that ignores these variables may misclassify disease or recommend treatment that is unsafe and/or unnecessary for that patient.

In aortitis, laboratory investigations support rather than confirm the diagnosis. Inflammatory markers are often raised in untreated inflammatory aortitis, but they are non-specific and may be normal in treated disease, isolated vascular disease or disease suppressed by IL-6 inhibition [9,44,51]. Additional laboratory abnormalities, such as anaemia, thrombocytosis, leucocytosis, renal impairment, or deranged liver function, may provide supporting context. Blood cultures should also be obtained when infection is suspected. Additional investigations, including serological testing for syphilis, tuberculosis, Q fever, fungal pathogens, hepatitis, and HIV, alongside autoimmune panels, complement levels, urinalysis, and IgG subclass quantification, should be guided by clinical phenotype rather than ordered routinely without clinical direction [1,7,31,32].

CTA often serves as the initial major imaging investigation owing to its speed, widespread availability, and capacity to define luminal anatomy, assess wall thickening, detect aneurysm or dissection, characterise calcification and thrombus, map branch-vessel involvement, and guide surgical planning [7,10]. While its strength lies in structural definition, its weakness is biological ambiguity. CTA, for example, can show that a wall is thickened or an aorta is aneurysmal, but it does not always distinguish active inflammation from scar, atherosclerosis, infection or postoperative change.

MRA is valuable in younger patients and for longitudinal surveillance because it avoids ionising radiation and can provide vessel wall information, including oedema and enhancement [10,52]. This is especially useful in Takayasu arteritis, where repeat imaging may be required over decades. However, MRI findings depend on protocol, timing, disease stage, treatment exposure and reader expertise. Persistent enhancement is not always equivalent to clinically meaningful active inflammation.

USS is central in suspected GCA, particularly for temporal and axillary artery assessment in experienced centres [10]. It is non-invasive, accessible and useful for cranial and extracranial branches. Limitations include operator dependence, restricted visualisation of deep thoracic and abdominal aortic segments, and inability to provide comprehensive whole-aorta assessment. In aortitis, USS should be treated as one part of a multimodality strategy rather than as a complete vascular assessment.

FDG-PET provides metabolic information and may identify active inflammation in the aorta and major branches, especially when systemic symptoms are prominent or the distribution of disease is unclear [10,44,45,46]. It may also detect occult malignancy or infection in selected contexts. However, FDG-PET interpretation remains challenging due to false-positive uptake from atherosclerotic plaque inflammation, concurrent infection, occult malignancy, postoperative changes, and technical factors affecting image acquisition and quantification. Low-grade uptake can be difficult to interpret, and treatment may reduce signal before structural risk has resolved.

Conventional angiography is now less central diagnostically because it visualises luminal change rather than vessel-wall inflammation and is invasive. It remains relevant when endovascular planning or intervention is required. Histopathology can be decisive when tissue is available, but it is usually obtained opportunistically after aortic surgery. Surgical specimens warrant careful examination for patterns of granulomatous, lymphoplasmacytic, suppurative, necrotising, or IgG4-rich inflammation, with microbiological stains and tissue cultures performed whenever infection remains a diagnostic consideration [26,27,31,32,53]. A pathology report showing aortitis should not close the case, but it should trigger clinical, microbiological, rheumatological and vascular imaging assessment.

Because many imaging findings are non-specific, a formal mimics checkpoint should be incorporated into the diagnostic pathway. Aortic wall thickening, periaortic soft tissue abnormalities, FDG uptake, and elevated inflammatory markers are non-specific findings that may arise from large-vessel vasculitis, infection, IgG4-related disease, malignancy, atherosclerotic inflammation, postoperative healing, retroperitoneal fibrosis, drug-induced injury, or concurrent overlapping processes [1,2,3,4,5,31,32,33,34,35]. A stronger diagnosis rests on concordance between phenotype, microbiology, serology, disease distribution, tissue findings where available and longitudinal behaviour. Discordance should prompt reassessment rather than automatic escalation of immunosuppression. The principal strengths, limitations and practical role of each modality are summarised in Table 2.

**Figure 3:**
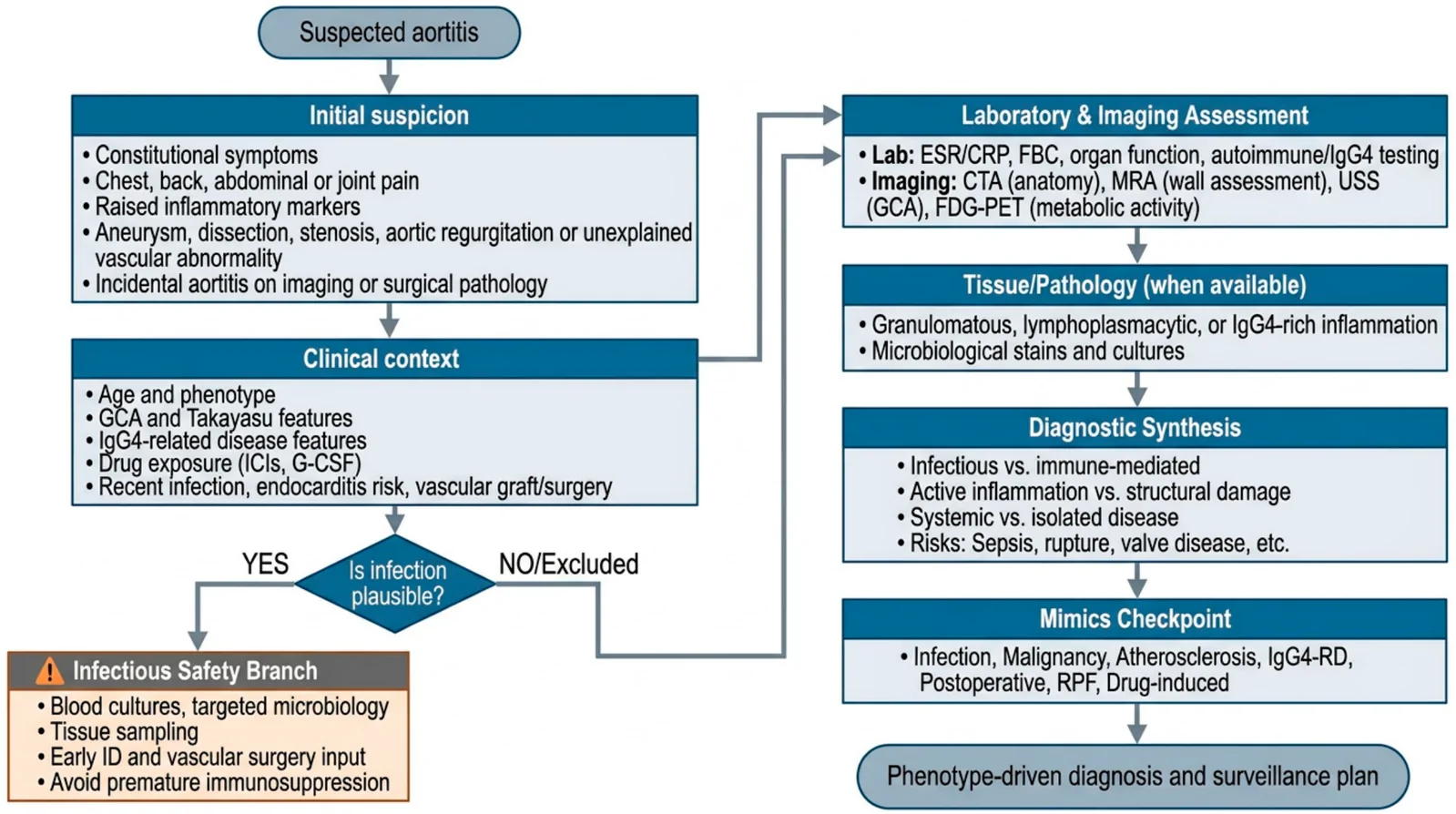
Diagnostic algorithm for suspected aortitis. The pathway emphasises clinical suspicion, infection exclusion, laboratory assessment, multimodality imaging, tissue assessment where available, and reassessment when clinical, microbiological, serological or imaging findings are discordant. Abbreviations: CRP, C-reactive protein; CTA, computed tomography angiography; ESR, erythrocyte sedimentation rate; FBC, full blood count; FDG-PET, fluorodeoxyglucose positron emission tomography; GCA, giant cell arteritis; G-CSF, granulocyte-colony stimulating factor; ID, infectious diseases; ICI, immune-checkpoint inhibitor; IgG4, immunoglobulin G4; IgG4-RD, IgG4-related disease; MRA, magnetic resonance angiography; RPF, retroperitoneal fibrosis; USS, ultrasound scan.

A practical diagnostic approach to suspected aortitis is proposed in Figure 3.

## 7. Management: Cause Before Escalation

Across all disease subtypes, treatment decisions should be individualised according to patient characteristics. A 70-year-old patient with diabetes, osteoporosis, and previous myocardial infarction should receive different glucocorticoid dosing and earlier steroid-sparing therapy than a younger patient without comorbidity. A patient with chronic kidney disease may not tolerate certain immunosuppressive agents. A patient with active malignancy receiving ICI requires oncology–rheumatology coordination to balance cancer control against vascular risk. Surgical candidacy depends not only on aneurysm size but on frailty, lung function, prior sternotomy, and tissue quality. These variables are part of safe and effective care, not secondary considerations.

Treatment should be guided by cause, activity, structural risk and patient vulnerability. A basic but often missed principle is that infectious aortitis must be actively considered before immunosuppression is escalated. When infection is suspected, management priorities include immediate broad-spectrum intravenous antimicrobials, prompt blood culture collection, pathogen-directed therapy once organisms are identified, and early vascular surgical evaluation [7,12,13]. In patients with aneurysm, pseudoaneurysm, dissection, rupture, persistent sepsis or high-risk anatomy, medical therapy alone is often insufficient. Contemporary aortic disease guidance supports open repair for infectious aortitis associated with aneurysm or dissection, while endovascular repair may be considered in selected high-risk patients, with prolonged antimicrobial therapy and suppressive treatment in selected unrepaired or recurrent cases [7,54].

A critical limitation is that negative microbiological cultures do not exclude infectious aortitis. Multiple factors can yield falsely negative cultures despite active infection, including prior antibiotic exposure, slow-growing organisms, graft-associated biofilms, and loculated periaortic abscesses with poor vascular communication. In patients with systemic sepsis, prosthetic material, saccular aneurysm, periaortic fluid, adjacent infection, immunosuppression or atypical imaging, source control and microbiological diagnosis should remain priorities even when inflammatory markers or blood cultures are initially negative [7,15,16,17].

For non-infectious large-vessel vasculitis, glucocorticoids remain the usual initial treatment for active disease. EULAR recommendations and the ACR/Vasculitis Foundation guideline support high-dose glucocorticoid induction in active GCA and Takayasu arteritis, followed by tapering according to disease control and toxicity risk [9,10,11]. While this traditional approach is effective, it comes at a cost. Prolonged glucocorticoid therapy is associated with substantial morbidity, increasing risks of infection, new-onset diabetes, hypertension, accelerated osteoporosis, cataract formation, metabolic weight gain, neuropsychiatric symptoms, progressive frailty, and compounded cardiovascular risk. In a disease defined by vascular vulnerability, treatment toxicity cannot be treated as a secondary issue.

Steroid-sparing agents have improved care in large-vessel vasculitis but do not eliminate long-term vascular risk. Tocilizumab increases sustained remission and reduces glucocorticoid exposure in GCA [41,55]. Among patients entering the GiACTA extension in remission after weekly tocilizumab, 42% remained tocilizumab- and glucocorticoid-free for a further two years [56]. Methotrexate has also been evaluated as adjunctive therapy, although the effect is more modest [57]. Adalimumab did not show clear steroid-sparing benefit in a randomised trial [58]. Translating trial outcomes into aortitis-specific protection requires caution because many trials focus on relapse, remission and steroid dose rather than late aneurysm formation, aortic dilatation, dissection, rupture or reintervention. Tocilizumab also suppresses CRP, making clinicians more dependent on symptoms, imaging and structured review [9,41,44,59].

Newer targeted therapies may expand treatment options. Mavrilimumab, which targets granulocyte-macrophage colony-stimulating factor receptor alpha, showed encouraging efficacy signals in a phase 2 trial in GCA [60]. Upadacitinib has shown efficacy in a phase 3 GCA trial when combined with a glucocorticoid taper [61]. By contrast, the phase 3 GCAptAIN trial found no statistically significant improvement in sustained remission at week 52 with secukinumab 300 mg plus a 26-week glucocorticoid taper compared with placebo plus a 52-week taper [62]. These developments signal a move from broad immunosuppression toward pathway-directed therapy. These agents should be interpreted cautiously, however, as it remains uncertain whether they prevent late aortic structural complications, perform consistently in extracranial large-vessel disease, or benefit clinically isolated aortitis.

The evidence base for Takayasu arteritis management remains limited compared to that for GCA. Glucocorticoids may induce remission, but relapse, progression and vascular complications are common [21,22,23]. Methotrexate, azathioprine, mycophenolate, tumour necrosis factor inhibitors and tocilizumab are used in practice, but the evidence base is weaker than in GCA and relies heavily on observational cohorts, small trials and expert consensus [9,11,23,63,64,65]. Surgery or endovascular treatment may be needed for critical stenosis, organ-threatening ischaemia, uncontrolled renovascular hypertension, aneurysm or dissection. Outcomes are generally better when active inflammation is controlled where feasible, but delay may allow irreversible organ injury. The practical tension is clear: intervene too early and restenosis or complications may follow but wait too long and vascular damage becomes fixed. Randomised evidence remains sparse: the TAKT tocilizumab trial did not meet its primary intention-to-treat endpoint, although sensitivity analyses suggested benefit, whereas abatacept did not prolong remission [42,43]. A small randomised open-label comparison of adalimumab and tocilizumab reported higher six-month remission with adalimumab, although its size and short follow-up limit inference [66]. These findings support cautious, individualised biologic use rather than assuming class-wide efficacy.

From a management perspective, clinically isolated aortitis poses difficult treatment decisions. Routine immunosuppression for every patient with histological aortitis after aortic surgery is not supported by strong evidence, but simple reassurance is unsafe [3,26,27]. A reasonable approach includes complete baseline imaging of the aorta and major branches, exclusion of infection and systemic inflammatory disease, cardiovascular risk optimisation, specialist vasculitis assessment and serial imaging. Immunosuppression should be individualised to evidence of persistent active inflammation, systemic disease, progression, high-risk anatomy or recurrent vascular lesions rather than applied reflexively. An illustrative, aetiology-specific surveillance framework — preferred modality, indicative interval and the strength of evidence behind each — is set out in Table 3.

**Figure 4:**
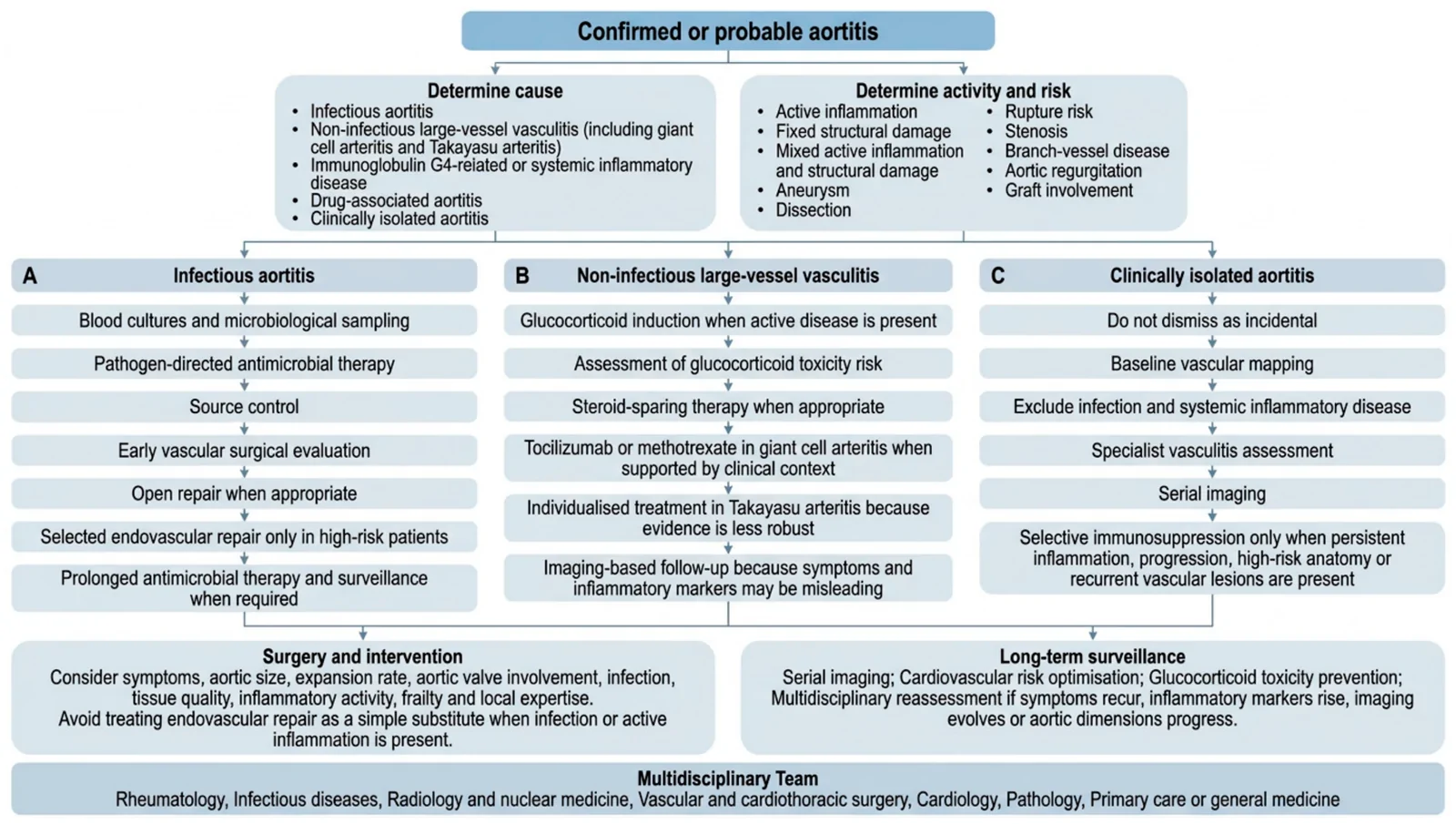
Management and follow-up pathway for aortitis. Treatment should be guided by aetiology, inflammatory activity, structural risk and patient vulnerability, with multidisciplinary reassessment and long-term imaging surveillance even after symptoms and inflammatory markers improve.

A cause-first management and follow-up pathway is outlined in Figure 4.

## 8. Surgery, Endovascular Repair and Timing

Aortitis requires clinicians to judge not only inflammatory activity but also the structural consequences of aortic disease. Multiple variables inform these decisions, including absolute diameter, expansion rate, symptomatic presentation, aortic valve involvement, dissection risk, branch-vessel ischaemia, infection status, patient frailty, and local surgical expertise. Inflammatory activity changes how these variables should be interpreted. An expanding aneurysm in active vasculitis differs fundamentally from stable degenerative disease, a pseudoaneurysm in infectious aortitis is not routine elective pathology, and stenotic lesions in Takayasu arteritis may restenose if treated during active inflammation.

Open surgical repair remains essential for many complex aortic complications, especially when tissue quality, infection, arch involvement, valve disease or extensive reconstruction is relevant. Endovascular therapy has changed the management of aortic disease and may reduce immediate physiological stress in selected patients. In inflammatory or infectious aortitis, however, endovascular repair should not be treated as a straightforward less-invasive substitute. It may be a bridge, a definitive option in selected high-risk patients or a hazard if infected tissue remains uncontrolled [7,12,13].

Every major aortic operation in which aortitis is suspected should also be treated as a diagnostic opportunity. Tissue should be examined carefully, and pathology should be linked to postoperative vascular medicine or rheumatology follow-up. Conversely, every unexpected pathology report showing aortitis should trigger reassessment rather than be treated as a minor operative finding. Care is strongest when the operating room, pathology department, imaging team, rheumatology service, infectious diseases team and long-term surveillance pathway are connected.

## 9. Multidisciplinary Care and Patient Communication

Multidisciplinary input must not dilute individual accountability. One named lead clinician should be documented at diagnosis and at every transition of care. For immune-mediated disease this will usually be the rheumatologist or vasculitis physician; for suspected or confirmed infection, infectious diseases and the aortic surgical team lead antimicrobial and source-control decisions; and for postoperative or structurally dominant disease, an aortic or cardiovascular specialist should manage surveillance. Local models may differ, but one clinician must integrate the evidence, communicate the plan and ensure follow-up. This is a pragmatic accountability model based on expert consensus rather than comparative trials [7,9,10,11].

The named lead synthesises clinical, microbiological, laboratory, imaging and pathological data with multidisciplinary input. Each review should record the working aetiology; infection status; activity, damage or both; dominant risk; agreed treatment; named owner and deadline for the next action; and date and modality of repeat imaging. Long-term follow-up remains with the named aortic, cardiovascular or vasculitis clinician until another service explicitly accepts a documented transfer.

Patients should be told that feeling well or having normal inflammatory markers does not end surveillance. The goals are to prevent aneurysm growth, dissection, rupture, ischaemia, repeated procedures, treatment toxicity and missed infection.

## 10. Evidence Gaps and Research Priorities

The first problem is definitional. Studies inconsistently distinguish between radiographically detected aortitis, histopathologically confirmed inflammation, clinically isolated disease, periaortic involvement, GCA-associated aortitis, Takayasu arteritis, IgG4-related disease, infectious aetiologies, and drug-induced injury [2,3,26,27,68]. Without shared definitions that specify aetiology, anatomical segment, inflammatory activity, structural damage and mode of detection, the literature will continue to produce results that are difficult to compare and hard to apply. The scale mismatch is concrete: inflammatory aortitis is reported in roughly 2–12% of thoracic aortic surgical series, with 41 cases among 1119 operations (3.7%) in one cohort; the largest established Takayasu cohort cited here included 318 patients, whereas the TAKT tocilizumab and abatacept trials enrolled only dozens of participants [22,38,42,43].

Monitoring remains a major weakness because symptoms, inflammatory markers and imaging each have important limitations when interpreted in isolation. Inflammatory markers are insufficient on their own, symptoms may be absent until complications occur, and imaging, although anatomically and metabolically informative, remains expensive and not always specific [2,3,10,44]. Composite activity scores in large-vessel vasculitis do not fully capture aortic-wall biology or reliably predict late aneurysm. The field needs biomarkers that reflect vascular inflammation more directly, imaging scores reproducible across scanners and readers, and endpoints that distinguish inflammatory relapse from structural progression.

Outcome selection also remains problematic as trials often measure what is feasible rather than outcomes most consequential to patients. While sustained remission, relapse prevention, and glucocorticoid dose reduction remain important, patients ultimately require protection from clinically meaningful complications: irreversible visual loss, progressive aneurysmal dilatation, aortic dissection or rupture, critical vascular stenosis, repeated interventions, accumulating disability, and premature mortality. These outcomes require longer follow-up, larger cohorts and registry-based collaboration. The rarity of aortitis makes research challenging, but it should not justify prolonged dependence on small retrospective series.

A further gap lies between surgical and immunology datasets. Surgical aortic cohorts may under-report inflammatory phenotype, immunosuppression, PET or MRI activity and long-term vasculitis outcomes. Vasculitis cohorts may under-report operative details, graft outcomes, restenosis, reintervention and pathology [26,27,53]. This separation highlights the need for multidisciplinary registries that capture both inflammatory control and structural aortic outcomes. Major aortitis studies should assess not only whether inflammation improves, but whether the aorta remains structurally safe.

Patient-centred outcomes remain under-represented in aortitis research, including persistent fatigue, medication adverse effects, psychological burden from aneurysm surveillance, claudication-related functional impairment, reproductive health concerns in younger patients and the cumulative toll of serial imaging. Similarly, quality of life, treatment satisfaction, and the quality of shared decision-making remain inadequately characterised. Aortitis is not only a vessel-wall disease. It is a long-term condition in which patients live with uncertainty about inflammation, structural progression, and future procedures. Future research should prioritise outcomes that matter to patients alongside traditional endpoints such as remission and aneurysm growth.

The literature also exposes several underdeveloped questions: the relative effectiveness of open versus endovascular techniques in infected aortic tissue, optimal antimicrobial duration stratified by pathogen and clinical response [7,14,54]; the capacity of FDG-PET to distinguish residual infection from sterile post-treatment inflammation [10,44,45,46,47]; consensus diagnostic criteria for IgG4-related aortitis [31,32,37]; systematic pharmacovigilance for ICI and G-CSF-associated disease [33,34,35]; and evidence-based surveillance intervals following surgically discovered clinically isolated aortitis [3,38,39,40]. These questions are clinically consequential because they determine whether patients receive timely source control and surveillance or move through care without a coherent plan.

Equity and access require separate attention because geographic and institutional variation affects access to vascular ultrasound, advanced imaging such as PET and MRI, specialised vascular medicine and rheumatology services, infectious diseases input and experienced aortic surgical teams. Patients diagnosed in centres without integrated pathways may receive delayed classification, inconsistent imaging and variable follow-up. Standardisation should not only improve academic comparability but it should reduce geographic and institutional variation in care.

## 11. Artificial Intelligence and Precision Medicine: Promise and Limits

Clinical evidence is preliminary but no longer entirely hypothetical. Duff et al. manually segmented the aorta on FDG-PET/CT in 50 patients with aortitis and 25 controls and tested 107 radiomic features in an AI-assisted diagnostic framework [69]. A subsequent multicentre study used convolutional-neural-network segmentation with separate training, test and external validation cohorts and reported validation area-under-the-curve values above 0.8 for several radiomic approaches [70]. In follow-up GCA imaging, a retrospective radiomics study trained 441 model combinations on 64 baseline scans; its selected random-forest model achieved an AUC of 0.92, but clinical follow-up testing involved only 19 scans [71].

These proof-of-concept studies remain limited by retrospective design, small and selected datasets, scanner and protocol heterogeneity, imperfect activity labels and limited prospective validation. Manual or model-dependent segmentation, overfitting, rare phenotypes, explainability and equity also constrain generalisability. No study has shown that algorithm-guided management improves vascular outcomes. AI should therefore support quantification and interval-change detection for multidisciplinary review, not autonomously decide infection, immunosuppression or surgery.

## 12. Conclusions

Modern imaging has made aortitis more visible, but not necessarily easier to classify. CTA, MRI, USS, FDG-PET and surgical pathology reveal disease that would previously have been missed. Visibility, however, has not solved classification, monitoring or treatment. The main task remains to determine whether aortic inflammation is infectious or immune-mediated, systemic or isolated, active or quiescent with residual vascular damage, and anatomically stable or structurally dangerous.

The practical priority is a documented, phenotype-specific plan that identifies the dominant risk, assigns responsibility to one named lead clinician and specifies the next clinical and imaging review.

Future progress should be judged by clinically meaningful outcomes: earlier diagnosis, fewer missed infections, lower cumulative glucocorticoid exposure, better prediction of aneurysm and dissection, fewer avoidable procedures, safer intervention and more equitable access to specialist imaging and expertise. Prospective cohorts and multidisciplinary registries should test whether the proposed framework improves these outcomes.

## Figures and Tables

**Table 1:** Practical comparison of giant cell arteritis and Takayasu arteritis.

Domain	Giant Cell Arteritis	Takayasu Arteritis
Typical demographic	Usually age ≥ 50 years; women are affected more often [19,20].	Usually begins at a younger age, with marked female predominance [21,22].
Vascular distribution	Cranial arteries, aorta and major branches; a large-vessel phenotype may lack cranial symptoms [24,25].	Aorta, arch branches, renal and other major arteries; stenosis or occlusion predominates, although aneurysm occurs [21,23].
Typical presentation	Headache, jaw claudication, visual symptoms, polymyalgia or constitutional illness; limb claudication or aortic disease may dominate.	Constitutional illness, pulse or blood-pressure asymmetry, bruits, limb claudication, renovascular hypertension or cerebrovascular symptoms.
Initial medical treatment	High-dose glucocorticoids; tocilizumab is the best-established steroid-sparing biologic [9,11,41].	Glucocorticoids plus a conventional immunosuppressant; biologics are considered for relapsing or refractory disease [9,11,42,43].
Evidence quality	Moderate-to-high for remission and glucocorticoid sparing; low for prevention of late aortic structural complications.	Predominantly observational; small randomised trials have produced mixed results.
Disease-specific consideration	Late thoracic aneurysm or dissection risk supports long-term structural surveillance [8,30].	HLA-B*52 is an association, not a diagnostic test; pregnancy requires pre-conception and multidisciplinary planning [28,29].

**Table 2:** Multimodality imaging in suspected or established aortitis.

Modality	Principal Strengths	Important Limitations	Practical Role
CTA	Rapid whole-aorta assessment; excellent lumen, calcification, thrombus, aneurysm, dissection and operative mapping.	Ionising radiation and iodinated contrast; wall thickening is biologically non-specific.	Common first structural study and main modality for acute complications or surgical planning [7,10].
MRI/MRA	No ionising radiation; vessel-wall oedema/enhancement and lumen can be assessed serially.	Longer acquisition, device/claustrophobia constraints and protocol-dependent activity interpretation.	Preferred for repeated surveillance in younger patients, particularly Takayasu arteritis [10,52].
USS	Accessible, repeatable and radiation-free; high value for temporal and axillary arteries in experienced hands.	Operator dependent; limited visualisation of deep thoracic and abdominal aortic segments.	First-line cranial/extracranial branch assessment in suspected GCA, not a complete whole-aorta map [10].
FDG-PET/CT	Maps metabolically active disease across the aorta and branches; may reveal occult infection or malignancy.	Atherosclerosis, infection, malignancy, treatment and postoperative change can confound uptake; radiation exposure.	Useful when activity or distribution is uncertain and for selected relapse questions [10,44,45,46].

**Table 3:** Pragmatic imaging-surveillance framework for aortitis. The intervals are illustrative rather than prescriptive and are not validated universal recommendations; they synthesise guideline principles, cohort evidence and expert practice. Timing should be individualised and shortened for new symptoms, active disease, uncertain infection or aortic growth.

Aetiology	Preferred Modality	Illustrative Interval (Individualise)	Evidence and Escalation Caveat
GCA	Baseline whole-aorta CTA or MRA; USS or FDG-PET for selected activity questions.	If aortic involvement or dilatation is present: repeat at 6–12 months, then every 6–24 months when stable.	Frequency is individualised; image earlier for symptoms, relapse concern or growth [7,10,30,67].
Takayasu arteritis	MRA for serial whole-aorta/branch assessment; CTA if MRA is unsuitable.	Active disease or treatment change: about 3–6 months; stable disease: 6–12 months, then individualised lifelong review.	No interval has been validated; do not equate normal markers with structural stability [9,10,11].
Clinically isolated aortitis	Baseline whole-aorta and major-branch CTA or MRA after pathology diagnosis.	Repeat at 6–12 months, then approximately annually if stable; continue lifelong structural surveillance.	Supported by recurrent-lesion and reintervention cohorts, not randomised trials [3,38,39,40].
IgG4-related aortitis	CTA or MRA of affected and adjacent vascular territories; assess other involved organs.	Reassess about 3–6 months after treatment, then approximately annually if stable.	Single-centre and retrospective evidence; accelerate for pain, inflammatory relapse or change in diameter [31,32,37].
Infectious aortitis	CTA for anatomy, complications and repair; FDG-PET/CT only for selected unresolved questions.	Early reassessment during treatment or after repair (often 2–6 weeks), then 3–6 months; annual imaging if graft or residual disease persists.	Very-low-certainty timing; the infectious-diseases/aortic team should individualise according to source control and anatomy [7,14,54,67].

## Data Availability

No new data were created or analysed in this study. Data sharing is not applicable to this article.

## Abbreviations

The following abbreviations are used in this manuscript:

ACR	American College of Rheumatology
AI	Artificial intelligence
CRP	C-reactive protein
CTA	Computed tomography angiography
ESR	Erythrocyte sedimentation rate
EULAR	European Alliance of Associations for Rheumatology
FBC	Full blood count
FDG-PET	Fluorodeoxyglucose positron emission tomography
G-CSF	Granulocyte-colony stimulating factor
GCA	Giant cell arteritis
GM-CSF	Granulocyte-macrophage colony-stimulating factor
HIV	Human immunodeficiency virus
HLA	Human leukocyte antigen
ICI	Immune-checkpoint inhibitor
ID	Infectious diseases
IFN-γ	Interferon gamma
IgG4	Immunoglobulin G4
IgG4-RD	Immunoglobulin G4-related disease
IL-17	Interleukin-17
IL-6	Interleukin-6
MHC	Major histocompatibility complex
MMP	Matrix metalloproteinase
MRA	Magnetic resonance angiography
MRI	Magnetic resonance imaging
PET	Positron emission tomography
RPF	Retroperitoneal fibrosis
TAK	Takayasu arteritis
Th	T-helper
TNF-α	Tumour necrosis factor alpha
USS	Ultrasound scan
